# The Role of Leptin in the Development of Energy Homeostatic Systems and the Maintenance of Body Weight

**DOI:** 10.3389/fphys.2021.789519

**Published:** 2021-12-10

**Authors:** Charles A. LeDuc, Alicja A. Skowronski, Michael Rosenbaum

**Affiliations:** Division of Molecular Genetics, Department of Pediatrics, Columbia University Irving Medical Center, New York, NY, United States

**Keywords:** leptin, adipose tissue, development, maintenance, energy balance

## Abstract

*LEP* is a pleiotropic gene and the actions of leptin extend well beyond simply acting as the signal of the size of adipose tissue stores originally proposed. This is a discussion of the multi-system interactions of leptin with the development of the neural systems regulating energy stores, and the subsequent maintenance of energy stores throughout the lifespan. The prenatal, perinatal, and later postnatal effects of leptin on systems regulating body energy stores and on the energy stores themselves are heavily influenced by the nutritional environment which leptin exposure occurs. This review discusses the prenatal and perinatal roles of leptin in establishing the neuronal circuitry and other systems relevant to the adiposity set-point (or “threshold”) and the role of leptin in maintaining weight homeostasis in adulthood. Therapeutic manipulation of the intrauterine environment, use of leptin sensitizing agents, and identification of specific cohorts who may be more responsive to leptin or other means of activating the leptin signaling pathway are ripe areas for future research.

## Introduction

In 1973, Coleman (1973) demonstrated that parabiosis of the obese (later *Lep*^ob^*)* mice with diabetes (later *Lepr^db^*) mice and wild type mice resulted in hypophagia and starvation of the *Lep^ob^* mice while not affecting the phenotype of the *Lepr^db^* mice. He postulated that “the obese mouse is able to produce sufficient satiety factor to regulate its food consumption, whereas the diabetes mouse produces satiety factor, but cannot respond to it”. Subsequently, Leibel and Hirsch (1984) and others (Welle et al., 1984) found that reduced body weight maintenance was accompanied by a decline in energy expenditure and an increase in hunger disproportionate to changes in body weight and composition that strongly resembled the metabolic state of the *Lep^ob^* and *Lepr^db^* mice. These observations were consistent with the so called “lipostatic” theory of body weight maintenance in which a “signal” reflecting adipose tissue mass affected hypothalamic neural circuitry regulating energy intake and expenditure (Kennedy, 1953; Mayer, 1955; Hetherington and Ranson, 1983).

The advent of large-scale genome-wide association studies (GWAS) combined with polygenic risk scoring has facilitated the identification of aggregate genetic factors determining body weight and the underlying energy homeostatic mechanisms that regulate it. By calculating an obesity propensity score from 2.1 million SNPs, individuals can be categorized into “obesity risk” deciles (Khera et al., 2019) with an overall correlation of genetic propensity score (GPS) and body mass index (BMI) of 0.29.

Needing 2.1 million SNPs to determine an obesity propensity score indicates that there are an almost uncountable number of minute genetic contributors that act in concert to determine a person’s genetic predisposition to adiposity. Mendelian breeding strategies, which can be employed in rodents, facilitate the identification of *critical* genetic variants. It is not surprising that the most important genes affecting energy homeostasis, including those first postulated by Coleman, are involved in the leptin-melanocortin pathway [including *Lep, Lepr, melanocortin 4 receptor (Mc4r), pro-opiomelanocortin (Pomc*)] were all first discovered in rodent models of extreme obesity and only subsequently identified in humans. Other critical feeding circuit genes in the same pathway were also first identified in animal models. *Npy* was discovered due to peptide abundance in porcine intestine extract, its co-localization in the murine brain (Tatemoto et al., 1982), and potent stimulation of energy intake following intracerebroventricular (i.c.v.) administration to rats (Clark et al., 1984). The orexigenic and thyroid releasing hormone (TRH) suppressive effects of *agouti-related peptide (Agrp)* were first recognized primarily because *Agrp* expression increased 10-fold in *Lep^ob^* mice (Manne et al., 1995).

It is now understood that *LEP* is a pleiotropic gene and that the actions of leptin extend well beyond simply acting as the signal of the size of adipose tissue stores originally proposed by Coleman. This is a discussion of the multi-system interactions of leptin with the development of the neural systems regulating energy stores, and the subsequent maintenance of energy stores throughout the lifespan. This review will attempt to show predominantly animal data regarding the prenatal and perinatal roles of leptin in establishing the neuronal circuitry of the adiposity set-point (or “threshold”) and the role of leptin as the signal regulating the “deployment” of those systems to maintain energy homeostasis in both humans and rodents.

## Leptin and the Ontogeny of Systems Regulating Body Weight

### Overview

The developmental environment of mice can be manipulated for prospective studies of weight regulation whereas human studies of the effects of obesogenic gene exposure are, of necessity, observational and epidemiological. Though data from rodent experiments are the most informative in elucidating the mechanistic developmental impact of leptin, there is compelling evidence indicating that both the development and regulation of body weight in humans reflects both pre- and post- natal gene × environment interactions.

### The Intrauterine Environment

The interactions between leptin and the developing neurocircuitry in rodents are dependent upon the nutritional environment in which they occur. The impact of maternal metabolic status and nutritional status of the pups in the prenatal and perinatal periods on body weight in adulthood can be directly investigated in rodents, and there have been numerous studies to this effect. Both maternal under- and over- nutrition during gestation affect these systems in a manner that favors the development of obesity (Breton, 2013). In general, offspring of dams who were malnourished during pregnancy show structural disorganization of the hypothalamic systems regulating appetite (Breton et al., 2009). Prenatal maternal undernutrition reduces the response of POMC neurons to energy status and food intake rhythm (Breton et al., 2009). Maternal overfeeding, especially with a high fat diet (see below), results in altered brain appetite regulators in the offspring (Rajia et al., 2010).

Rodent models of perinatal undernutrition include maternal caloric or protein restriction during gestation and/or lactation (Vickers et al., 2000; Yura et al., 2005; Delahaye et al., 2008; Cripps et al., 2009) and increasing litter size to lower milk availability per pup in a litter (Aubert et al., 1980; Marangon et al., 2020). Perinatal maternal undernutrition drastically reduces the postnatal surge of plasma leptin, disturbing particularly the hypothalamic wiring as well as the gene expression of the anorexigenic POMC neurons in male rat pups (Delahaye et al., 2008).

The macronutrient composition of gestational undernutrition and the perinatal environment interact in their effects on adult rodent adiposity. While the association of gestational undernutrition by caloric restriction induces only a small degree of increased weight gain in the adult offspring (Lagisz et al., 2014), the evidence is stronger that specifically restricting protein in pregnant dams results in increased body weight of the offspring which is exacerbated by HFD exposure (Ozanne et al., 2004; Lagisz et al., 2014; Juan De Solis et al., 2016). Caloric or protein restriction during the suckling period and rearing in large litters has an opposite effect in that the offspring gain less weight during the first postnatal weeks and this lower weight persists in these pups throughout lifetime (Ozanne et al., 2004; Patterson et al., 2010).

There is strong evidence that maternal HFD feeding during perinatal period as well as overnutrition of sucking pups (by decreasing litter size) increases body weight and predisposes the offspring to greater weight gain when exposed to HFD in adulthood (Ainge et al., 2011; Lagisz et al., 2015; Ribaroff et al., 2017). Some of the leptin-related consequences of overfeeding in rodents are related to the magnitude of the postnatal leptin surge (Marangon et al., 2020; Skowronski et al., 2021), leptin sensitivity in the CNS (Kirk et al., 2009), neuroanatomy of the leptin-dependent feeding circuits (Kirk et al., 2009; Vogt et al., 2014), and epigenetic changes—specifically hypermethylation of the hypothalamic POMC promoter (Plagemann et al., 2009; Marco et al., 2014).

In humans, epigenetic studies have examined the effects of the intrauterine environment, primarily in the form of factors affecting DNA methylation, histone acetylation, and expression of microRNAs, on gene expression relevant to obesity and its co-morbidities. Increased DNA methylation decreases the transcription of relevant genes and is affected by parental obesity, maternal diet (e.g., nutrition, folic acid content, and other methyl donors), gestational diabetes (see below), maternal medications (antibiotics and antipsychotics), smoking, and exposure to chemicals such as bisphenol (Inadera, 2013; Reynolds et al., 2013). Major intrauterine environmental influences on the risk of subsequent obesity in the offspring via these processes and others include maternal adiposity and gestational weight gain, under- and over- nutrition, maternal stress, and various chemicals, pharmaceuticals, etc. to which the mother and fetus may be exposed to during pregnancy are summarized in Table 1.

**TABLE 1:** Overview of intrauterine epigenetic factors relevant to subsequent adiposity.

Prenatal variable	Effect
Maternal pre-partum weight and weight gain during pregnancy	In studies comparing siblings born to the same mothers before and after bariatric surgery, the infants developing in the weight reduced, post-bariatric surgery environment show lower adiposity, blood pressure, circulating concentrations of insulin, gene expression relevant to diabetes, autoimmune disease, and vascular disease risk (Guenard et al., 2013). Weight gain during pregnancy has a strong positive correlation with birthweight and the incidence of subsequent childhood obesity (Oken et al., 2009). These correlations are augmented 2–5 fold in mothers with pre-partum obesity compared to those who were neither overweight nor obese prior to pregnancy
Intrauterine nutritional and chemical environment	Maternal diet (overall nutrition, low folate, and low amounts of other methyl donors), diabetes mellitus, use of steroids, antipsychotics or antibiotics, smoking, exposure to chemicals such as bisphenol all alter DNA methylation of genes that favor increased subsequent adiposity (Inadera, 2013)
Prenatal undernutrition	Maternal undernutrition or compromised fuel delivery to the fetus [e.g., placental dysfunction) are all associated with increased risk of intrauterine growth retardation (small for gestational age, SGA)] and with subsequent obesity and acquisition of co-morbidities at lower levels of body fatness (Barker, 1997; Ravelli et al., 1998; Yarbrough et al., 1998; Hattersley and Tooke, 1999; Moore et al., 1999; Godfrey and Barker, 2000) depending upon the timing of intrauterine undernutrition (Ravelli et al., 1976). It has been hypothesized that early intrauterine malnutrition might affect development of hypothalamic feeding circuits while the anti-obesity effects of perinatal malnutrition might be due to suppression of adipocyte formation
Prenatal overnutrition	Prenatal overnutrition is exemplified by the infant of the mother with diabetes (usually gestational) with high ambient glucose. It is difficult to separate the metabolic effects of gestational diabetes and those of maternal adiposity in this population. Gestational diabetes is associated with an increased risk of obesity in the offspring, independent of the degree of maternal obesity (Pettitt et al., 1983, 1987, 1988).
Maternal stress during pregnancy	Metabolic (e.g., obesity, diabetes, undernutrition, and illness), psychiatric (e.g., depression, anxiety, and bereavement), or pharmacological (e.g., steroids, antidepressants, and antibiotics) maternal stressors have all been associated with increased risk of offspring obesity via effects on developing neural systems regulating energy homeostasis, endocrine systems affecting risk of diabetes–including increased activity of the hypothalamic-pituitary-adrenal (HPA) axis, immune system alterations resulting in increased circulating concentrations of pro-inflammatory cytokines, decreased concentrations of adiponectin relative to fat mass, and increased risk of hypertension (Entringer et al., 2012; Entringer and Wadhwa, 2013)

Leptin and its signaling pathways figure prominently in these intrauterine and perinatal systems affecting the development and maintenance of energy stored as adipose tissue. Leptin is an adipocyte-derived hormone providing a long-term signal to the CNS regarding quantity of stored body adiposity, largely by binding to the long form of leptin receptor, LepRb, in POMC and AgRP/NPY) neurons. These first order neurons are primarily located in the arcuate nucleus of the hypothalamus (ARH) and are well described in mediating neuroendocrine systems related to energy homeostasis (Schwartz et al., 2000). Activation of the Jak2/STAT3 pathway by leptin signaling increases the activity of POMC neurons and the expression of *Pomc* and *Cart* (Cocaine and amphetamine-related transcript) while inhibiting the orexigenic AgRP/NPY neurons and decreasing the expression of *Agrp* and *Npy* (Hahn et al., 1998; Cowley et al., 2001). POMC is posttranslationally cleaved by proconvertases (PC1 and PC2) and other peptidases to create several smaller peptides including β-endorphin and α-melanocyte stimulating hormone (α-MSH) (Benjannet et al., 1991). α-MSH inhibits energy intake and stimulates energy expenditure via melanocortin 4 receptors (MC4R) and, to a lesser degree, melanocortin 3 receptors (MC3R) located on second order neurons (Seeley et al., 1997; Sweeney et al., 2021). AgRP is an antagonist of MC4R (Ollmann et al., 1997) and opposes the effects of POMC. NPY is an agonist of the NPY receptors which mediates additional orexigenic effects (Schwartz et al., 2000). Mutations in the *POMC* gene lead to severe human obesity (Krude et al., 1998) while rodent *Pomc* knockouts are obese and less sensitive to leptin (Challis et al., 2004). While congenital mouse *AgRP* knockouts have a limited metabolic phenotype (Qian et al., 2002) with normal body weight, adiposity, and food intake, conditional ablation of AgRP neurons in adulthood induces an ultimately lethal anorexia (Luquet et al., 2005).

In addition to adipose tissue, leptin is produced by the placenta (Hassink et al., 1997) and stomach (Mix et al., 1999) in humans. In embryonic mice, leptin is also produced by hair follicles, liver, heart, bone, and cartilage (with both protein and mRNA detected) (Hoggard et al., 2000). During pregnancy in humans, circulating leptin is increased by 1.5–3 fold in the second and third trimesters of pregnancy (Butte et al., 1997; Hardie et al., 1997; Highman et al., 1998; Sattar et al., 1998), followed by a significant drop in the early postpartum period, as early as 24 h after delivery (Sivan et al., 1998; Lage et al., 1999). Leptin is detected in fetal circulation as early as 19 weeks of gestation with a rapid increase in fetal leptin between weeks 33 and 41 and correlates with fetal size (Butte et al., 1997). At delivery, newborn plasma leptin correlates with gestational age and birthweight (Cetin et al., 2000) with subsequent decreases to low levels within the first few days after parturition (Schubring et al., 1999).

### Leptin and Development of the Neural Circuitry Regulating Body Weight

**FIGURE 1:**
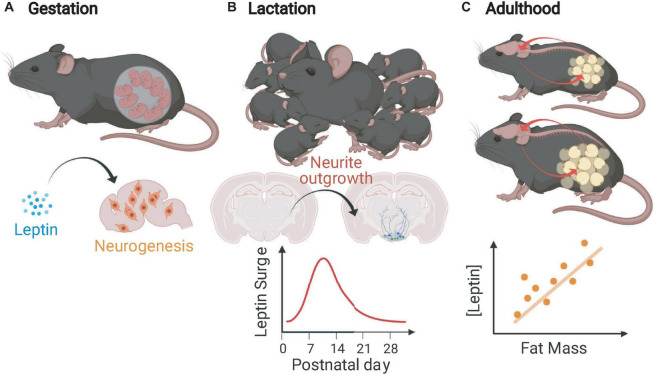
Leptin functions in mice during gestation, lactation, and adulthood. **(A)** In the gestational period, leptin mediates neurogenesis and proliferation of other brain cells (not limited to gestation). **(B)** During the immediate postnatal (lactational) period, mice undergo a leptin surge that is critical for the outgrowth of projections from feeding circuit essential neurons. **(C)** In adult mice, leptin is produced in rough proportion to stable fat mass, informs the CNS about energy stores, and protects against fat loss (Figure created with BioRender.com).

The changing functions of leptin with regards to neurogenesis and adipose tissue from conception to adulthood in rodents are illustrated in Figure 1. Since the fetal intrauterine environment receives a continuous transplacental supply of glucose and other nutrients necessary for growth, leptin does not regulate “appetite” in the traditional sense *in utero*, but such wide expression of *Lep* could have other functions. The complete impact of prenatal leptin on energy homeostatic and other systems is not fully understood, but there is clear evidence that it acts as a neurogenic factor *in utero*. Compared to wild type mice, mice that lack leptin have brains that are both smaller by weight and have reduced cortical volume (Bereiter and Jeanrenaud, 1979), have fewer cells at embryonic day 16 (E16) and E18, and fewer recently born cells at E14 and E16 in the neuroepithelium. Intracerebroventricular leptin injection of E14 *Lep^ob^* embryos normalized the number of neuroepithelium cells at E16 (Udagawa et al., 2006). The neurogenic effects of leptin are still evident postnatally. Intraperitoneal administration of leptin daily for 2 to 4-week-old *Lep^ob^* mice resulted in increased dry brain weight due, at least partially, to an increase in cell number as indicated by total brain DNA which increased at a greater rate than brain weight (Steppan and Swick, 1999).

Deficiency of leptin during development impairs the formation of the feeding circuits; the density of projections originating from the ARH to other hypothalamic regions critical in energy homeostasis [including the paraventricular nucleus (PVH), the dorsomedial hypothalamic nucleus (DMH), and the lateral hypothalamic area (LHA)] is decreased (Bouret et al., 2004). Ahima et al. (1998) first demonstrated that mice undergo a postnatal leptin surge during which the circulating leptin concentration is significantly higher than predicted by fat mass and is unrelated to amount of stored energy (Bouret et al., 2004). The timing of the leptin surge is critical; it overlaps temporally with the generation of homeostatic feeding circuits in the hypothalamus and brain stem (Bouret et al., 2004; Biddinger et al., 2020). Studies in *Lep^ob^* mice directly indicate that leptin acts as a neurotrophic factor during the perinatal period. Administration of exogenous leptin to *Lep^ob^* mice to mimic the naturally occurring leptin surge (P4–P12) rescues the axonal density of feeding circuit neurons while supplementation of leptin in adult *Lep^ob^* mice fails to restore these hypothalamic projection densities (Bouret et al., 2004). In addition to the hypothalamus, the nucleus of the solitary tract (NTS) within the brain stem is critical in energy homeostasis, especially for the integration of the viscerosensory signals. The majority of *Glp-1* expressing neurons within the NTS express *LepRb* and project primarily to the PVH. The density of GLP-1 innervation from the NTS to the PVH is augmented in Lep*^ob^* mice indicating leptin’s role in the development of this circuit (Biddinger et al., 2020).

Leptin influences astrocyte development. There is a marked increase in glia cell number between postnatal weeks 2 and 3 in rodents (Bandeira et al., 2009) coinciding with the natural leptin surge (Ahima et al., 1998). Astrocytes express the long form of the leptin receptor (*LepRb*) (Pan et al., 2008; Kim et al., 2014). Exogenous leptin administration between P8 and P12 increases the proliferation of astrocytes in the hypothalamus. This is a direct effect of leptin since proliferation is decreased when *LepRb* is conditionally removed from these cells (Rottkamp et al., 2015). Conditional deletion of *LepRb* in adult mouse astrocytes leads to glial morphological changes and increased synaptic inputs onto hypothalamic POMC and AgRP neurons (Kim et al., 2014). These mice also show diminished leptin-regulated feeding suppression, suggesting a direct impact of leptin on astrocyte development and function in adult mice (Kim et al., 2014) which is supported by studies demonstrating leptin-mediated neurogenesis post-stroke (Avraham et al., 2013) as well as a model of Alzheimer’s disease (Calio et al., 2021) in rodents.

### Effects of Perinatal Leptin on Subsequent Adiposity

In rats and mice (Marangon et al., 2020; Skowronski et al., 2021), maternal high fat diet (HFD) feeding during gestation and/or lactation, or overfeeding the pups via reduced litter size augments the postnatal leptin surge (see below) and subsequent weight in the offspring. Caloric or protein restriction of dams or underfeeding the pups by increased litter size reduces and delays the leptin surge with a reduction in weight into adulthood (Delahaye et al., 2008). Skowronski et al. (2021) demonstrated that all pups undergo a postnatal leptin surge, but in the underfed state, the surge is delayed and transitory. Excess or deficiency in leptin does not affect body weight and adiposity of pups prior to the second week of life. There is no difference in body weight or composition between *Lep^ob^* mice, hyperleptinemic, and wild type mice at postnatal day 10 (Mistry et al., 1999). Unlike adult mice with mature feeding circuits, in the first 2 weeks of mouse life, leptin does not influence feeding, instead, it is critical in the proper formation of these circuits.

In addition to changes in magnitude and timing of the leptin surge, maternal diet influences the development of neurocircuitry relevant to energy intake. HFD feeding during pregnancy in rodents is associated with disruptions in the normal patterns of projections in the hypothalamic feeding circuits, including decreased AgRP immunoreactive fibers in the PVH (Kirk et al., 2009; Vogt et al., 2014) and reduced density of α-MSH projections from the ARH to PVH, DMH and LHA in 8-week old progeny (Vogt et al., 2014). These are the same projections disrupted in congenitally leptin deficient mice suggesting that effects of maternal diet on the weight of the offspring may be mediated through effects on the postnatal leptin surge which, in turn, alters the development of the feeding circuitry.

Elevated circulating leptin concentrations during the rodent suckling period leads to increased adiposity and weight gain in adult offspring when exposed to obesogenic diets (Vickers et al., 2008; Skowronski et al., 2020). In leptin transgenic TET-ON mice, oral doxycycline (DOX) causes leptin overexpression in proportion to the concentration of DOX, allowing for the transient elevation of leptin without increasing fat mass and consequently avoiding any obesity co-morbidities. These leptin transgenic mice were transiently exposed to elevated leptin during the first 3 weeks of life (to mimic the augmented leptin surge induced by postnatal overfeeding) and in adulthood were metabolically identical to control littermates which were not postnatally supplemented with excess leptin. However, when the adult mice were exposed to a HFD challenge, the postnatally hyperleptinemic mice gained more fat mass and weight than littermate controls and the difference in body weight gain was statistically significant within 3 days (Skowronski et al., 2020). Since this experiment isolated the augmented leptin surge from the other confounds of postnatally overfeeding pups, it suggests that leptin, in isolation, can reprogram the body weight set point such that mice are less sensitive to future increases in leptin. Male rat offspring given exogenous leptin IP during the first 2 weeks of life resulted in increased diet-induced weight and fat gain in adulthood (de Oliveira Cravo et al., 2002; Vickers et al., 2008). Interestingly, Sanchez et al. (2005) administered leptin orally to postnatal pups to investigate the role of leptin in breastmilk. This oral leptin was absorbed by the immature gastric epithelium of the neonate and down-regulated endogenous leptin production in the pup, suggesting leptin’s potential role in the short-term control on food intake during the lactation period. Additionally, orally-fed leptin to postnatal pups (P0–P20) had the opposite effect of IP leptin injections—the leptin-fed offspring gained less weight in adulthood and had a lower preference for fat-rich foods when exposed to HFD (Pico et al., 2007) compared to their controls suggesting that postnatal oral leptin may permanently reduce endogenous leptin production and lead to increased responsiveness to leptin in adult rats.

## Leptin and the Physiology Feeding Behavior, Energy Expenditure, Neuroendocrine Function, and Autonomic Function in Adulthood

### Overview

Humans and mice stably maintain body energy stores (fat) without conscious effort to adjust food intake or energy expenditure. Adult humans, regardless of adiposity, gain weight at an average of approximately 0.3–0.5 kg/year (Zheng et al., 2017) (∼3,000 kcal stored energy) between the ages of 18–55 years in females and 21–55 years in males, while ingesting over 800,000 kcal/year (Ford and Dietz, 2013) thereby suggesting the operation of homeostatic mechanisms for body weight regulation. Hyperphagia and hypometabolism (including decreased circulating concentrations of bioactive thyroid hormones and sympathetic nervous system tone and increased parasympathetic nervous system tone) act together to favor weight regain after successful weight reduction and oppose efforts by most individuals to sustain weight loss (Leibel and Rosenbaum, 2010; Thomas et al., 2014; Hall and Kahan, 2018). The level of body energy stores that are “defended” in a given individual depends upon homeostatic systems—the structure and function of which are, at least partially, determined by early leptin exposure. As discussed above, the degree and timing of leptin exposure reflects a number of key factors in the intrauterine environment including maternal health and nutrition.

The effects of leptin on energy homeostasis are not limited to the development of these systems. Leptin serves as a marker of adipose tissue stores and energy balance and is a major signal governing the extent to which individuals respond to attempts to lose weight and keep it off.

### Energy Homeostasis and Weight Loss

**FIGURE 2:**
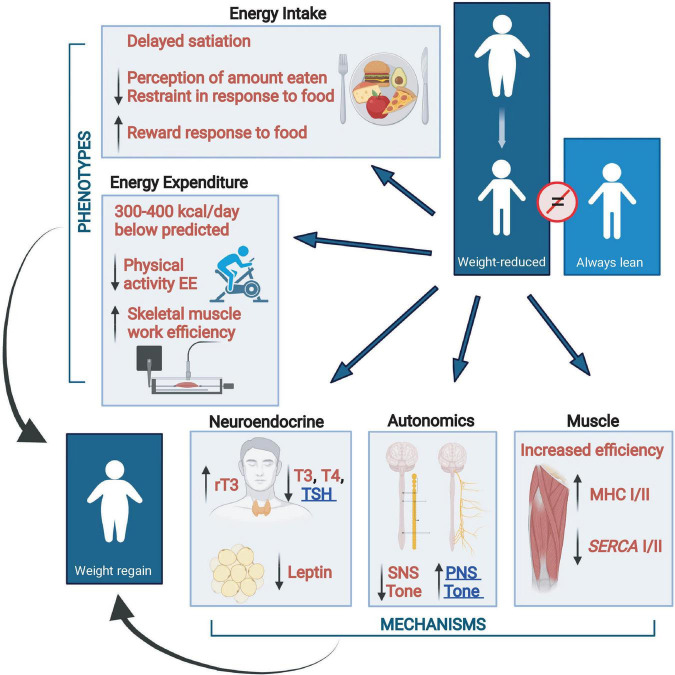
Changes from baseline in energy balance and homeostatic systems during maintenance of a 10% or greater reduced body weight and their responsiveness to exogenous leptin in individuals who initially had obesity or never had obesity (Rosenbaum and Leibel, 2014). Energy expenditure due to physical activity is calculated as the difference between direct measurement of 24-h energy expenditure and measurement of resting energy expenditure plus diet-induced thermogenesis. Eating behavior, including energy intake, is examined by visual analog scales during a fixed liquid formula meal, kcal of the liquid formula consumed to reach satiation, and by fMRI studies of brain responses to food. Assessments of autonomic nervous system activity were made by analyses of heart rate variability during sequential blockade of the parasympathetic and sympathetic nervous systems with atropine and esmolol, respectively, and by 24-h urine catecholamine excretion. Skeletal muscle contractile efficiency was measured by graded bicycle ergometry. Myosin heavy chain (*MHC*) and sarcoplasmic endoplasmic reticulum Ca^++^-dependent ATPase (*SERCA*) muscle gene expression studies were done by mRNA quantification in biopsies of vastus lateralis muscle. All phenotypes opposing sustained weight loss are responsive to leptin repletion except for PNS tone and TSH which are underlined in blue. SNS, sympathetic nervous system; PNS, parasympathetic nervous system; T3, triiodothyronine; T4, thyroxine; rT3, reverse T3; TSH, thyroid stimulating hormone; MHC, myosin heavy chain; SERCA, sarcoplasmic endoplasmic reticulum Ca^++^-dependent ATPase (Figure created with BioRender.com).

There are changes in energy intake and expenditure which act concordantly to oppose weight loss and the maintenance of reduced body weight. Changes related to energy intake include increased, hunger, delayed satiation, increased neuronal responses to food in the orbital frontal cortex and areas related to food reward and decreased responses in the prefrontal cortex and areas related to food restraint. Homeostatic changes in energy expenditure are due, at least in part, to changes in skeletal muscle via increased work efficiency due to increased expression of the more efficient myosin heavy chain I (MHC) and sarcoplasmic endoplasmic reticulum Ca^++^-dependent ATPase 2 (SERCA2). This hypometabolic state is augmented by changes in neuroendocrine function (decreased circulating concentrations of bioactive thyroid hormones and leptin) and, at least during reduced weight maintenance, increased parasympathetic and decreased sympathetic nervous tone (Larrouy et al., 2008; Leibel and Rosenbaum, 2010; Rosenbaum and Leibel, 2014; Dulloo and Schutz, 2015). The multi-system interactions that oppose the maintenance of a reduced body weight are summarized in Figure 2.

### Leptin and the Activity of Energy Homeostatic Systems

As noted above, body fat stores, are regulated by multiple systems that conspire to defend energy stores (fat) against energy imbalance by adjusting energy intake and output to maintain a relatively constant level of available energy over time. Leptin provides a signal to the brain regarding the quantity of fat stores as well as energy balance (weight loss in particular). The result is that the intensity of the leptin signal, and the energy homeostatic responses to changes in that intensity, are determined by both the ambient leptin concentration (Myers et al., 2008) and by the nutritional state of the organism. It is notable that leptin mediates the development of the same brain regions that ultimately influence neuroendocrine functions, autonomic efferents, and food-related behaviors (Korner et al., 1999, 2001; Korner and Aronne, 2003).

Leptin-mediated signals regulate a complex neural system that mediates what is physiologically apparent as the regulation of body weight via the integration of short- (e.g., gut-derived hormones and glucose) and longer- (e.g., leptin, insulin, and free fatty acids) term signals related to energy homeostasis (Korner et al., 1999, 2001; Schwartz et al., 2000; Korner and Aronne, 2003). Teleologically, these body weight regulatory systems should be biased toward “defending” against sustained weight loss which could threaten reproductive capacity/fertility and/or survival (Rosenbaum and Leibel, 1998). It is not surprising that the effects of leptin administration show a strong functional bias in favor of the preservation of body fat stores versus their reduction as discussed below.

Shortly after leptin was cloned from the *Lep^ob^* mouse (Zhang et al., 1994), humans with the nonsense mutations in the *LEP* gene were reported (Montague et al., 1997). Similar to mice, humans that are leptin deficient have morbid obesity and reduced muscle mass, are hyperphagic, and hypometabolic. When leptin deficient humans (Farooqi et al., 1999) or mice (Hwa et al., 1997) are supplemented with leptin, they reverse these phenotypes. Short term administration of leptin to lean, *Lep^ob^*, or to diet-induced obese mice reduces appetite, body weight, and adiposity (Campfield et al., 1995; Halaas et al., 1995; Pelleymounter et al., 1995). The treatment of leptin in these initial studies was short (Pelleymounter et al., 1995) (from two successive 5-day treatments to 28 days at the longest) but the effects were striking; *Lep^ob^* mice were normalized and lean mice reduced body fat from 12.2 to 0.7% (Halaas et al., 1995).

The leptin receptor is highly expressed in the cells of the hypothalamic nuclei–the development of which is mediated by leptin (discussed above). These nuclei play prominent roles in homeostatic weight regulation (Pan and Mg Myers, 2018) as well as communicate with other telencephalic and diencephalic neurons that mediate behavioral responses to food. Many neurons outside the hypothalamus also express the receptor, though their functional roles in these cells remain unclear.

### Leptin Signaling Is Dependent Upon the Nutritional Environment

As discussed above, the interactions of leptin with the developing neurocircuitry are dependent upon the nutritional environment in which those circuits develop. Similarly, the effects of leptin on energy homeostatic systems are dependent upon the nutritional environment (fat mass and energy balance) in which leptin is administered. Most of these systems are more sensitive to leptin following weight loss (especially during maintenance of reduced weight) compared to initiation of weight loss or during dynamic weight loss; and these energy homeostatic responses are stronger to leptin depletion than excess (Leibel, 2002; see Table 1).

The discovery of leptin was initially expected to ameliorate the obesity epidemic; however, this expectation has never been met. Unlike mice, administration of exogenous leptin to humans with or without obesity has little or no effect on body weight even at grossly supraphysiological doses (Heymsfield et al., 1999). Leptin administration to individuals during caloric restriction (negative energy balance), with concomitant declines in circulating leptin concentrations, results in a small reduction in appetite but no significant changes in energy expenditure or neuroendocrine function (Hukshorn et al., 2000, 2002, 2003).

Leptin administration does not seem to induce or perpetuate weight loss in humans. Heymsfield et al. (1999) administered leptin in placebo, physiological, and supraphysiological doses to 54 lean and 73 obese subjects for 4 weeks; and to 47 obese subjects for 24 weeks. Participants with obesity were placed on diets restricting caloric intake to about 500 kcal/day below usual, but dietary compliance was not assessed and there was no significant weight loss in the placebo group. After 4 weeks, overall weight reduction in leptin-treated subjects with or without obesity was not different from placebo-treated. Participants with obesity, not those without obesity, who received the highest doses of exogenous leptin (sufficient to raise circulating leptin concentrations more than 20-fold above initial) for a period of 24 weeks showed a small significant weight loss (2.3 kg more than placebo) and a small but statistically insignificant decrease in daily energy intake. The high circulating leptin concentrations and low levels of weight loss in participants with obesity following exogenous leptin administration have been interpreted to indicate “leptin resistance” (Friedman and Halaas, 1998; Kalra, 2001; Lee et al., 2001; Scarpace and Zhang, 2007). This conclusion that leptin resistance at usual weight reflects leptin resistance only in individuals with obesity is not supported by the data since individuals without obesity were not more responsive to leptin. If anything, these data would suggest that any individual at usual weight is likely to be leptin resistant.

Similarly, Moon et al. (2011), found no significant effects of 10 mg BID of subcutaneous leptin administration to 71 weight stable participants with obesity and type 2 diabetes managed by diet alone. Mackintosh and Hirsch (2001) reported no effects of high dose (0.3 mg/kg/day) leptin administration—the highest dose used by Heymsfield et al. (1999)—on autonomic nervous system (ANS) tone in weight-stable lean subjects which is significant compared to the significant effect of leptin repletion on sympathetic tone noted following weight loss.

The lack of effects of leptin on weight loss induction or potentiation, even in supraphysiological doses, are in stark contrast to the potent effects of leptin in weight-reduced individuals who are weight stable (i.e., in energy balance) (see Figure 2). Simple leptin repletion to levels present prior to weight loss in this population “reverses” most, if not all, of the metabolic and behavioral phenotypes that oppose the reduction of energy stores. These include the increased hunger and delayed satiation as well as the hypometabolism and its components (increased muscle efficiency, decreased sympathetic nervous system tone and circulating concentration of bioactive thyroid hormones) that favor weight regain (Rosenbaum et al., 2005, 2008b,a; Kissileff et al., 2012; Hinkle et al., 2013). Taken together, these data suggest that the major function of leptin in energy homeostasis is to signal the organism that energy stores are low and/or that energy intake is inadequate to maintain weight. The individual is much more responsive to a drop in circulating concentrations of leptin below a personalized leptin threshold than to a rise in leptin above that threshold.

### Leptin Signaling Is Non-linear

Circulating leptin concentrations are determined by fat mass (cell number x size) but the relationship is modified downward (decreased leptin per unit of fat mass) at reduced weight and even more so during negative energy balance during weight loss (Halle et al., 1999). The actions of some hormones, such as the effects of insulin on glucose utilization, are linear within a physiological range (Rizza et al., 1981). However, leptin actions are non-linear and most consistent with a “threshold model” wherein leptin effects are triggered when leptin levels fall below a certain individualized “set-point” and leptin response is attenuated if administered when circulating concentrations are above that point. The “threshold” for sufficiency of leptin action for any individual is determined by genetic, developmental and environmental factors that influence both the structure of relevant parts of the CNS as well as the acute responses of those cells to ambient leptin. Individuals who have been obese have higher thresholds and more leptin (fat mass) is needed to create a state of sufficiency in the CNS. Once that level is achieved, further increases in circulating leptin concentrations have little physiological effect.

When fat mass is reduced by caloric restriction, a fall in circulating leptin concentrations below the threshold “informs” specific neurons that invoke behavioral (hunger) and metabolic (reduced energy expenditure) changes that act coordinately to restore body fat (leptin). This individual molecular-cellular leptin threshold does not decrease with weight loss or with prolonged maintenance of reduced weight (Rosenbaum et al., 2008a); hence, the metabolic/behavioral response to reduced leptin does not abate. The threshold is an individualized molecular-cellular phenotype such that response to declines in leptin below the threshold are similar regardless of initial adiposity. What differs between individuals with and without obesity is the threshold. Therefore leptin signal transduction is dependent upon an individual’s “usual weight” and any ongoing or previous negative energy balance (Morabito et al., 2017).

Response to exogenous leptin is also dependent on energy stores and balance. When leptin concentrations are raised above an individual “threshold” (which is higher in individuals with obesity), the relevant neuronal tracts are less sensitive (LeDuc and Leibel, 2019; Zhao et al., 2019) and further leptin administration evokes little if any response in humans (Rosenbaum et al., 2018b). For reasons not yet understood, when leptin concentrations are reduced below the threshold and the individual is in negative energy balance (caloric restriction) the effect of leptin repletion is small (Hukshorn et al., 2000, 2002, 2003). However, in low leptin states where there is little energy imbalance, such as reduced weight maintenance, congenital leptin deficiency, or lipodystrophy, most, if not all, of the metabolic and behavioral effects of low leptin are at least partially relieved (Farooqi et al., 1999; Oral et al., 2002; McDuffie et al., 2004; Rosenbaum et al., 2005, 2008b,b; Park et al., 2007; Kissileff et al., 2012; Hinkle et al., 2013; Brown et al., 2018).

The threshold model described above presumes that the primary function of leptin is to preserve fat mass in times of perceived undernutrition in defense of preserving reproductive integrity and survival of a species. This is supported by the effects of leptin repletion in states of hypothalamic amenorrhea which can be caused by excessive exercise or decreased food intake and leads to infertility and bone loss. Short term treatment with leptin from 3 months to 36 weeks recovers menstruation and corrects the abnormalities in the gonadal, thyroid, growth hormone, and adrenal axes (Welt et al., 2004; Chou et al., 2011). This is consistent with the role of leptin acting as a starvation signal; the drop in circulating leptin concentration signals the gonadal system to decrease procreation when energy stores are scarce to prevent pregnancy that would be overly challenging to both the mother and fetus.

### Implications for Future Research

The critical roles played by leptin in the early development of systems regulating body weight and its subsequent actions within those symptoms has implications for the prevention and treatment of obesity. Obesity risk could theoretically be altered via modification of the development of leptin-mediated neuronal circuitry regulating body weight toward the defense of a lower body weight in those at risk. Manipulation of the intrauterine environment by diet or other means to reduce fetal overnutrition or undernutrition may reduce the propensity toward later obesity (Delahaye et al., 2008; Breton et al., 2009; Breton, 2013). Though it is unlikely that leptin will be effective as a weight loss medication – its efficacy might be increased in the setting of leptin sensitizing agent (Ravussin et al., 2009) or in individuals with disproportionately low levels of leptin (Ahima, 2008). The potent effects of leptin after weight loss indicates the need for longer term studies of the effects of leptin on the likelihood of successful reduced weight maintenance.

A critical question is whether the leptin threshold is changeable, particularly in a downward direction. While there are clearly genetic effects on human adiposity, and while the majority of these genes are expressed in the central nervous system, the precise manner(s) in which these effects are integrated is not yet clear (Loos, 2018). The threshold model is consistent with the phenomenology of the similarity of responses to weight loss among obese and non-obese individuals. In humans the phenotypes associated with increased metabolic efficiency and drive to eat do not abate with time (Shick et al., 1998; DelParigi et al., 2007; Rosenbaum et al., 2008a), suggesting that prolonged maintenance of reduced body weight can only be achieved by indefinite attention to both food intake and exercise (Wing and Hill, 2001). However, it is possible that, in addition to the effects of allelic variants, there are developmental effects on energy homeostatic circuits that can influence an individual’s apparent threshold for minimum body fat that could potentially be modified through earlier intervention in those “at-risk” for subsequent adiposity. An example of this potential to manipulate the “set-point” prenatally is the reduction in fatness, blood pressure, circulating concentrations of insulin and gene expression relevant to diabetes, autoimmune disease, and vascular disease in children who develop in a post-bariatric surgery intrauterine environment compared to their siblings who were gestated prior to surgical weight loss in the mother (Guenard et al., 2013).

## Conclusion

Leptin is a highly pleiotropic molecule that influences the prenatal and perinatal development of the major neuronal tracts that determine the body weight that is “defended” over a lifetime. Subsequently, it provides the primary signal that determines the activity of the same regulatory systems.

Leptin, importantly, functions primarily as a signal of decreased energy stores and/or negative energy balance from the periphery to the CNS. In the state of negative energy balance, the resulting rapid decrease in circulating leptin concentration is sensed by the CNS and, in response, drives hunger, suppresses energy expenditure, and reduces reproductive competence; despite potentially underlying obesity (Boden et al., 1996). Similar effects are seen as a result of a lesser degree of relative hypoleptinemia following weight loss at which time most individuals are quite responsive to leptin repletion. This “predictive” characteristic of leptin production by adipose stores acts to increase fat mass, and thereby protect reproductive integrity in times of undernutrition, is arguably the leptin function that is most important in an evolutionary context. Going forward, leptin therapy during adulthood is likely to be a factor in the maintenance of reduced weight after successful treatment by weight loss.

## Author Contributions

CL, AS, and MR contributed equally to the preparation of this manuscript and approved the submitted version.

## Conflict of Interest

The authors declare that the research was conducted in the absence of any commercial or financial relationships that could be construed as a potential conflict of interest.

## Publisher’s Note

All claims expressed in this article are solely those of the authors and do not necessarily represent those of their affiliated organizations, or those of the publisher, the editors and the reviewers. Any product that may be evaluated in this article, or claim that may be made by its manufacturer, is not guaranteed or endorsed by the publisher.
